# Supplementary material for: Positive selection on human gamete-recognition genes
Source: PeerJ. 2018 Jan 11;6:e4259. doi: 10.7717/peerj.4259 (PMC5767332; doi:10.7717/peerj.4259)
Supplement: Supplemental Information 4 — Each table shows the number of synonymous or nonsynonymous polymorphic sites within humans (in the 1000 Genomes Project phase 1), and the number of synonymous or nonsynonymous fixed differences between humans and a single chimpanzee haplotype (accession numbers for chimpanzee genes are given in the main text). The chi-squared value for each two-by-two contingency table, and the associated P-value, are shown for each gene. [file peerj-06-4259-s004.docx]

Appendix Table ##: McDonald-Kreitman tests of balancing selection.

| Gene | Polymorphisms (within humans) | Fixed differences (human-chimpanzee) | Total |
| --- | --- | --- | --- |
| *C4BPA* | | | |
| Synonymous | 10 | 6 | 16 |
| Nonsynonymous | 17 | 17 | 34 |
| Total | 27 | 23 | 50 |
| χ^2^= | 0.684 | | |
| *P*= | 0.408 | | |
| *ZP3* | | | |
| Synonymous | 7 | 1 | 8 |
| Nonsynonymous | 19 | 3 | 22 |
| Total | 26 | 4 | 30 |
| χ^2^= | 0.006 | | |
| *P*= | 0.935 | | |
| *ZP2* | | | |
| Synonymous | 12 | 6 | 18 |
| Nonsynonymous | 21 | 4 | 25 |
| Total | 33 | 10 | 43 |
| χ^2^= | 1.761 | | |
| *P*= | 0.184 | | |
